# Canine *Staphylococcaceae* circulating in a Kenyan animal shelter

**DOI:** 10.1128/spectrum.02924-23

**Published:** 2024-01-11

**Authors:** Hatice Akarsu, Anne M. Liljander, Anna Lacasta, Paul Ssajjakambwe, Isabelle Brodard, Jérémy D. R. Cherbuin, Sergi Torres-Puig, Vincent Perreten, Peter Kuhnert, Fabien Labroussaa, Joerg Jores

**Affiliations:** 1 Institute of Veterinary Bacteriology, University of Bern, Länggassstrasse, Bern, Switzerland; 2 SIB Swiss Institute of Bioinformatics, Écublens, Switzerland; 3 Animal and Human Health Program, International Livestock Research Institute, Nairobi, Kenya; 4 Department of Veterinary Pharmacy, Clinical and Comparative Medicine, College of Veterinary Medicine, Animal Resources and Biosecurity, Makerere University, Kampala, Uganda; 5 Multidisciplinary Center for Infectious Diseases, University of Bern, Bern, Switzerland; 6 Graduate School for Biomedical Science, University of Bern, Bern, Switzerland; Texas A&M University, College Station, Texas, USA

**Keywords:** *Staphylococcaceae*, *Staphylococcus aureus*, *Staphylococcus pseudintermedius*, *Mammaliicoccus sciuri*, dog, resistance, virulence gene, nose, wound infection, Africa, Kenya

## Abstract

Animal shelters, especially in resource-poor countries, bring together pets from different regions and with different backgrounds. The crowding of such animals often results in infectious diseases, such as respiratory infections. This study characterized *Staphylococcaceae* from diseased and apparently healthy dogs housed in an animal shelter in Kenya, to determine their antibiotic resistance profiles, their genetic relatedness, and the presence of dominant clones. Therefore, bacteria were collected from all 167 dogs present in the shelter in June 2015 and screened for *Staphylococcaceae* using standard cultivation techniques. In all, 92 strains were isolated from 85 dogs and subsequently sequenced by PacBio long-read sequencing. Strains encompassed nine validated species, while *S. aureus* (*n* = 47), *S. pseudintermedius* (*n* = 21), and *Mammaliicoccus (M.) sciuri* (*n* = 16) were the three most dominant species. Two *S*. *aureus* clones of ST15 (CC15) and ST1292 (CC1) were isolated from 7 and 37 dogs, respectively. All 92 strains isolated were tested for their antimicrobial susceptibility by determining the minimum inhibitory concentrations. In all, 86 strains had resistance-associated minimal inhibitory concentrations to at least one of the following antimicrobials: tetracycline, benzylpenicillin, oxacillin, erythromycin, clindamycin, trimethoprim, kanamycin/gentamicin, or streptomycin. Many virulence-encoding genes were detected in the *S. aureus* strains, other *Staphylococcaceae* contained a different set of homologs of such genes. The presence of mobile genetic elements, such as plasmids and prophages, known to facilitate the dissemination of virulence- and resistance-encoding genes, was also assessed. The unsuspected high presence of two *S*. *aureus* clones in about 50% of dogs suggests dissemination within the shelter and a human source.

**IMPORTANCE:**

Microbiological data from sub-Saharan Africa are scarce compared to data from North America, Europe, or Asia, and data derived from dogs, the man’s best friend, kept in sub-Saharan Africa are largely missing. This work presents data on *Staphylococcaceae* mainly isolated from the nasal cavity of dogs stationed at a Kenyan shelter in 2015. We characterized 92 strains isolated from 85 dogs, diseased and apparently healthy ones. The strains isolated covered nine validated species and we determined their phenotypic resistance and characterized their complete genomes. Interestingly, *Staphylococcus aureus* of two predominant genetic lineages, likely to be acquired from humans, colonized many dogs. We also detected 15 novel sequence types of *Mammaliicoccus sciuri* and *S. pseudintermedius* indicating sub-Saharan-specific phylogenetic lineages. The data presented are baseline data that guide antimicrobial treatment for dogs in the region.

## INTRODUCTION

The dog, man’s best friend, is the oldest domesticated animal witnessing mankind’s cultural evolution (1). Dogs often live in close association with humans and positively impact human health in terms of psychological welfare and physical health due to increased interactions with other people and physical activity, respectively (2). The total number of dogs worldwide is estimated to be around 700 million and is likely to rise in the future partly due to increasing wages in currently low- and middle-income countries (LMIC). The number of stray dogs, broadly referring to unowned or community-owned dogs, is expected to follow the same trend already causing major public health issues especially in LMIC (3) but not restricted to (4). In contrast to the health benefits provided by pet dogs, stray dogs contribute to environmental pollution, and dog bite incidence, and can act as reservoirs of many important zoonotic pathogens such as rabies virus (5), *Leptospira* (6), and *Capnocytophaga canimorsus* (7). The canine microbiome, mainly inhabiting the mucosal and skin surfaces, is poorly characterized compared to the human microbiome. However, the advent of sequencing techniques revealed that dogs harbor a huge diversity of microbial species, which can widely differ between dogs with respect to their health status or environmental conditions (8, 9). As dogs explore their habitat rather through scents, they tend to expose their nose to different objects in their environment. Therefore, the composition of the upper airway microbiota was shown to be less conserved than the lower airway counterpart (10). More than 20 different phyla were identified in the nasal cavity of healthy dogs (11) with proteobacteria such as *Moraxella* spp. or *Ralstonia* spp. predominantly detected (8, 12). Different members of both coagulase-positive and coagulase-negative *Staphylococcaceae* (13), including *Staphylococcus aureus*, *Staphylococcus pseudintermedius* (14, 15), and *Mammaliicoccus sciuri* (16), can also be regularly isolated from dogs and humans. *S. aureus* is often associated with wound- and surgery-associated infections but also with pyoderma and otitis, while *S. pseudintermedius* is an opportunistic pathogen causing frequently canine ear and skin infections among others. These species have been implicated in human and animal disease and are known to harbor different sets of virulence factors (15) and resistance genes, including methicillin resistance (17, 18). Methicillin-resistant *Staphylococcus aureus* (MRSA) and *Staphylococcus pseudintermedius* (MRSP) have been isolated from companion animals including cats, dogs, and horses (19) but also from livestock species. A recent study performed on Australian shelter dogs identified at least one *Staphylococcus* spp. in ~75% of the sampled dogs, of which 10%–20% were methicillin-resistant (11). Numerous reports confirmed the zoonotic potential of several *Staphylococcaceae* species (18, 20), which can pose a risk to animal- and human health, especially when they are multi-drug resistant (MDR). A better understanding of the potential reservoirs in different regions of the world and the circulation of MDR *Staphylococcaceae* is definitively needed. While such information is progressively acquired especially in developed countries, there is a lack of information in other parts of the world including the African continent (21).

Animal shelters are a melting pot giving home to animals with different health statuses and backgrounds, including stray dogs. Such animal shelters could represent an adequate environment to study the diversity of bacterial strains circulating in a particular region. In this study, we phenotypically and genotypically characterized 92 *Staphylococcaceae* strains isolated mainly from the nasal cavity of dogs kept together in an animal shelter in Nairobi, Kenya. First, we assessed the diversity of the isolated strains using matrix-assisted laser desorption ionization–time of flight (MALDI-TOF) mass spectrometry followed by PacBio sequencing. The obtention of closed high-quality genomes allowed us to define their repertoires of antimicrobial resistance genes and virulence traits. Such a study does not only provide baseline data for comparison to other *Staphylococcaceae* strains but also provides insight into the resistance and virulence genes that may be present in *Staphylococcaceae* from dogs in this region.

## RESULTS

### Strains of this study encompassed nine validated species of *Staphylococcaceae*

Out of the 167 dogs sampled, we isolated 92 strains from 85 different dogs mainly from nasal swabs collected in the framework of a routine clinical sampling in an animal shelter in Nairobi, Kenya. In all, 14 strains (15% of the total) were isolated from clinically affected dogs presenting nasal discharge and/or signs of emaciation; three of them being kept in a dedicated isolation unit. All remaining strains were isolated from apparently healthy dogs and the metadata are compiled in Dataset S1. In all, 59 strains (64% of the total) showed a beta-hemolytic phenotype. The initial species designation, based on MALDI-TOF MS analysis and phenotyping using the VITEK2 Gram-Positive (GP) card (Dataset S1), highlighted the presence of at least nine validated species belonging to the genera *Staphylococcus* and *Mammaliicoccus*. Among the *Staphylococcus* (*S*.) species analyzed, *S. aureus* was most represented (*n* = 47), followed by *S. pseudintermedius* (*n* = 21), *S. cohnii* (*n* = 1), *S. haemolyticus* (*n* = 1), *S. saprophyticus* (*n* = 1), and *S. nepalensis* (*n* = 1). The *Mammaliicoccus* (*M*.) species encompassed *M. sciuri* (*n* = 16), *M. lentus* (*n* = 2), and *M. vitulinus* (*n* = 2). The strains isolated from animals with clinical symptoms were restricted to *S. aureus* (*n* = 9), *S. pseudintermedius* (*n* = 3), *S. cohnii* (*n* = 1) and *M. sciuri* (*n* = 2).

### Genome sequencing revealed many plasmids and new MLST sequence types of *M. sciuri* and *S. pseudintermedius*

**Fig 1:**
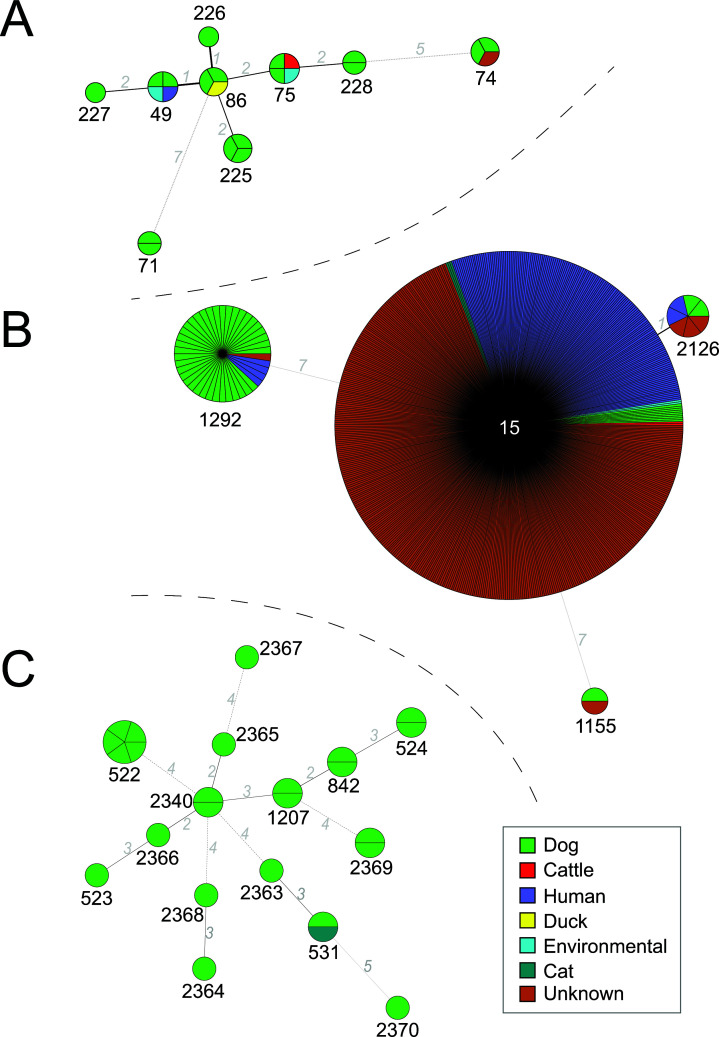
Minimum spanning trees of three *Staphylococcaceae* specie*s* with respect to their host origin. Minimum spanning tree (MST) based on multilocus sequence typing (MLST) data were built based on (**A**) 23 *Mammaliicoccus sciuri* strains, (**B**) 611 *Staphylococcus aureus* strains, and (**C**) 25 *Staphylococcus pseudintermedius* strains. The MLST data were downloaded from PubMLST and the trees were built with Bionumerics v8.1.1. The sequence type (ST) numbers are displayed in black or white, while gray numbers indicate allele differences between the STs. The host origin is displayed using the color code depicted in the legend.

All *Staphylococcaceae* strains were sequenced using PacBio long reads (average length 10.4 kbp). Their chromosomes were assembled and circularized with high coverage values ranging from 144× to 941× (Dataset S1). Genome data per strain including the closed chromosome and plasmids—if present—have been deposited at NCBI (https://www.ncbi.nlm.nih.gov/), project number PRJNA942599. The main features associated with these chromosomes are summarized in Table 1; Fig. 1; Fig. S1. In addition, 60 out of 92 strains sequenced (65% of the total) had one or more circularized plasmids ranging from small 2- to 5-kbp rolling-circle replicative (RCR) plasmids up to a 76.6-kbp conjugative plasmid found in *S. saprophyticus* (Fig. S2A; Dataset S1). Several large (i.e., ~20 to 50 kbp) nonconjugative plasmids were also identified. Interestingly, all *S. aureus* strains carry at least one plasmid except for the ST1155 strain, in which we did not detect one. Conversely, a plasmid was found in only five *M*. *sciuri* (30%) and six *S*. *pseudintermedius* (29%) strains and none was detected in the two *M. vitulinus* and *M. vitulinus*-like strains. Many plasmids were found to carry antimicrobial resistance genes (described in the “Phenotypic antimicrobial resistance and resistance-encoding genes” section below and Dataset S1) but also to encode bacterial mobile genetic elements (MGEs). We detected the presence of several insertion sequences (IS) of the IS*6* family, often associated with transposon (Tn) sequences belonging to the Tn*552*-like family (Dataset S1). In addition, several mobilization sequences including the origin of transfer (*ori*T) sequences and relaxase genes were identified. Two plasmids, carrying a complete multigene mobilization system (i.e., *mobCAB* and *ori*T) characteristic of the plasmid pC221, were isolated in *M. lentus* and *S. nepalensis* (Dataset S1). The presence of prophage sequences was investigated using PHASTER. All strains had at least a prophage sequence, while complete prophage sequences were detected in more than 60% of the strains (Fig. S2B).

**TABLE 1:** Ninety-two canine *Staphylococcaceae* strains analyzed in this study

Species (No of strains analyzed)	Resistance to at least one antimicrobial	Chromosome size (bp)	Chromosomal GC% content	MLST sequence types detected
*M. lentus* (2)	2	2,839,622 ± 29,750	32 ± 0.01	N/A
*M. sciuri* (16)	16	2,818,085 ± 50,527	32.55 ± 0.05	ST49 (2), ST71 (1), ST74 (2), ST75 (2), ST86 (2), ST225 (3), ST226 (1), ST227 (1), ST228 (2)
*M. vitulinus-like* (1)	0	2,567,438	33.11	N/A
*M. vitulinus* (1)	0	2,688,133	32.76	N/A
*S. aureus* (47)	45	2,772,257 ± 17,051	32.91 ± 0.01	ST15 (7), ST1155 (1), ST1292 (37), ST2126 (2)
*S. cohnii* (1)	1	2,569,178	32.43	N/A
*S. haemolyticus* (1)	1	2,526,786	32.82	ST8 (1)
*S. nepalensis* (1)	1	2,877,688	33.21	N/A
*S. pseudintermedius* (21)	17	2,595,637 ± 53,005	37.65 ± 0.09	ST522 (5), ST523 (1), ST524 (2), ST531 (1), ST842 (1), ST120 (1), ST2340 (1), ST2363 (1), ST2364 (1), ST2365 (1), ST2366 (1), ST2367 (1), ST2368 (1), ST2369 (2), ST2370 (1)
*S. saprophyticus* (1)	1	2,601,396	33.25	N/A

MLST profiling of the *M. sciuri*, *S. aureus*, *S. pseudintermedius,* and *S. haemolyticus* was carried out using established MLST schemes accessible in PubMLST (Dataset S1). New alleles and sequence types (STs) were added to the PubMLST database. The 16 *M. sciuri* strains isolated in this study belonged to nine different STs including four novel STs, namely ST225 (*n* = 3), ST226 (*n* = 1), ST227 (*n* = 1), and ST228 (*n* = 2). The other *M. sciuri* strains belonged to ST49 (*n* = 2), ST74 (*n* = 2), ST75 (*n* = 2), ST86 (*n* = 2), and ST71 (*n* = 1). Interestingly, strains belonging to the established ST49, ST75, and ST86 clustered together with strains isolated from cattle, ducks, and cats, mainly isolated in Asia (Fig. 1A; Fig. S1A). Unexpectedly, one *M. sciuri* strain of ST49 from the PubMLST data set was isolated from the urine of a human patient in the Czech Republic.

All *S. aureus* strains belonged to already published ST types, including ST15 (*n* = 7), ST2126 (*n* = 2), ST1155 (*n* = 1), and ST1292 (*n* = 37) (Fig. 1B). All these STs also encompassed strains isolated from different hosts and geographic locations even if human strains are largely overrepresented (Fig. 1B and S1B). We investigated their phylogenetic relationship using core genome data (Fig. S3). Apart from the strain of ST1155, the remaining *S. aureus* strains could be grouped into two clusters: ST15/ST2126 (CC15) and ST1292 (CC1). It is therefore likely that most of the *S. aureus* strains originated from two clones circulating in the shelter at the time of the sampling. To explore this possibility further, we built a minimum spanning tree of all African isolates deposited in pubMLST between 2013 and 2017, independently of their host (Fig. S4). This analysis allowed us to identify a human isolate (ST4707, CC1) closely associated with all our *S. aureus* ST1292 isolates (Fig. S4A). In addition, the results also show that all our *S. aureus* ST15 isolates tend to cluster preferentially with human isolates (Fig. S4B). No clear association nor origin was found for the *S. aureus* ST1155 isolate (Fig. S4C).

The 21 *S*. *pseudintermedius* strains analyzed in this study were represented by 15 different MLST profiles. Among those, 11 novel STs (ST2363 to ST2370 and ST522 to ST524) were deposited, each containing one strain except ST2369, ST522, and ST524 comprising 2, 5, and 2 strains, respectively (Fig. 1C). All these STs were dog-specific with the sole exception of ST531, which contained a *S. pseudintermedius* strain isolated in an apparently healthy cat in Poland in 2016.

The only strain of *S. haemolyticus* isolated in this study belonged to the ST8. This ST type primarily contained only methicillin-resistant *S. haemolyticus* strains of human origin isolated in Japan (*n* = 4) and the UK (*n* = 1) apart from a strain recently isolated from a cat in Brazil.

### Phenotypic antimicrobial resistance and resistance-encoding genes

**Fig 2:**
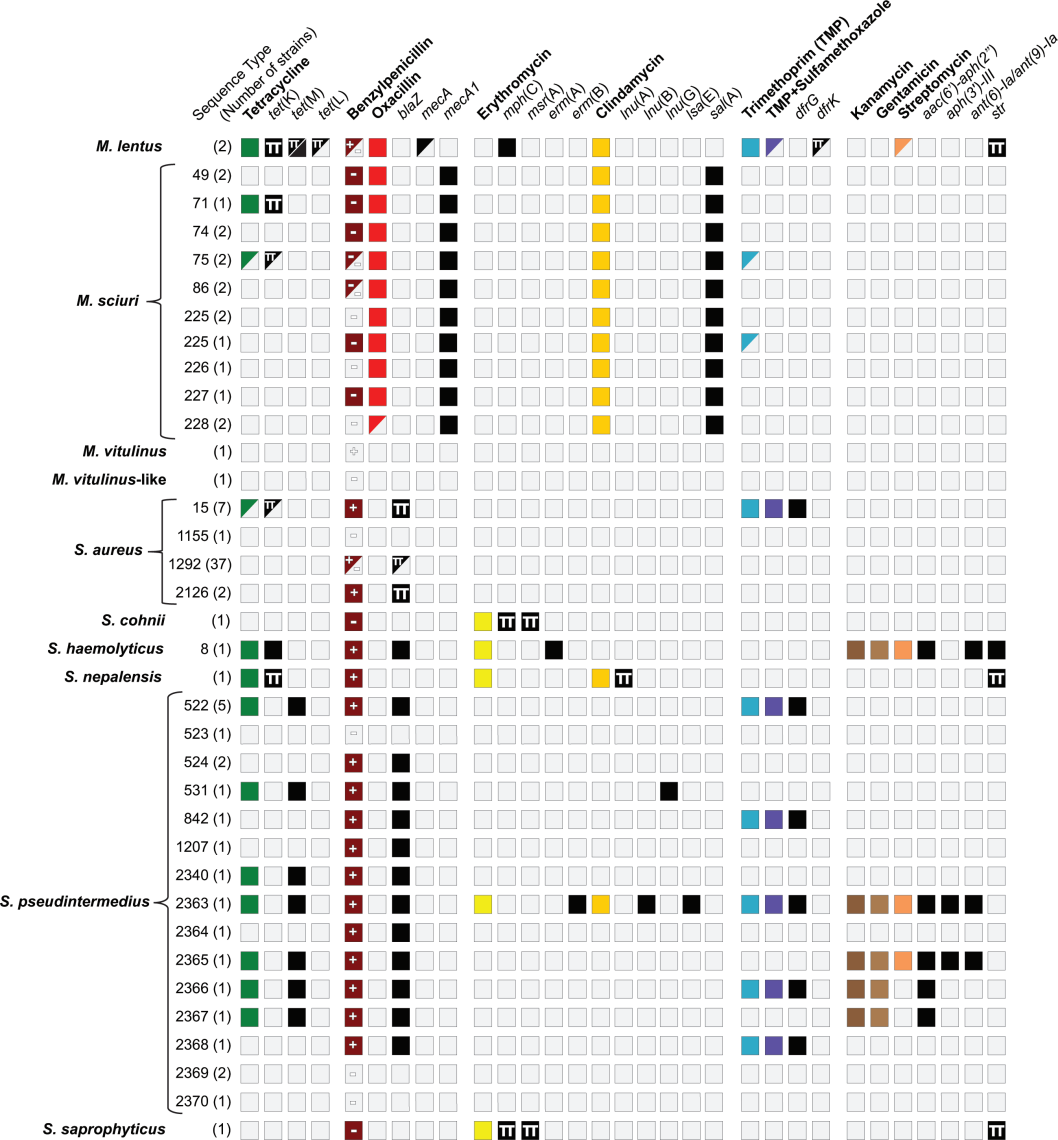
Phenotypic antimicrobial resistance and presence of resistance genes of the canine *Staphylococcaceae* strains of this study. Antimicrobial susceptibility was determined by minimum inhibitory concentration (MIC) testing. Antimicrobials are indicated in bold followed by the gene(s) associated with the resistance. Colored boxes represent strains associated with resistant phenotypes for the corresponding antibiotics (MICs values used can be found in the Methods section). Black-filled boxes represent the presence of resistance genes in the bacterial genome. Boxes in light gray represent susceptible phenotypes or the absence of the corresponding resistance genes. Half-filled boxes indicate that the phenotype was only found in a subset of strains belonging to the same ST. Plus (+) and minus (−) signs correspond to the positive and negative results of the nitrocefin tests performed on all strains. The greek letter Pi indicates that the gene is plasmid-encoded.

We determined the antimicrobial resistance profiles of all 92 strains against a wide range of antimicrobials. Results are summarized in Fig. 2 and displayed in detail in Dataset S1.

Overall, tetracycline resistance was observed in 21 strains (23% of the total). Resistance was associated with the presence of the tetracycline efflux pump-encoding gene *tet*(K) in *M. sciuri* (*n* = 2; ST71 and ST75), *M. lentus* (*n* = 2), *S. aureus* (*n* = 4; ST15), *S. nepalensis* (*n* = 1), and *S. haemolyticus* (*n* = 1; ST8). Except for the latter, the *tet*(K) gene was always found on a small ~4.4 kbp pT181-like plasmid. The only noticeable exception was found in *M. lentus* Dog026, which not only carried the *tet*(K) gene on a large ~26 kbp plasmid but also carried two additional plasmids of ~17 kbp and 6 kbp encoding the *tet*(M) and *tet*(L) genes, respectively (Dataset S1). Tetracycline resistance in *S. pseudintermedius* (*n* = 11) was restricted to the presence of a chromosomal copy of the *tet*(M) gene. Interestingly, two copies of the *tet*(M) gene were found in the five *S*. *pseudintermedius* strains belonging to the ST522.

Methicillin-resistant *S. aureus* and *S. pseudintermedius* strains were not detected (Fig. 2). In *S. aureus*, resistance to beta-lactams was restricted to the presence of a plasmid-encoded *blaZ* gene in all resistant strains (*n* = 45, 96%) (Fig. 2). Strains belonging to the ST2126 and ST15 carried the *blaZ* gene on a ~21 kbp pMW2-like plasmid (22). By contrast, the *blaZ* gene in ST1292 strains (*n* = 36) was encoded on a plasmid with similarity to the plasmid pWBG762 (23). A chromosomal *bla*Z gene was present in all resistant *S. pseudintermedius* (*n* = 17, 81%) and *S. haemolyticus* (*n* = 1, 100%) strains. Resistant phenotypes were also reported for benzylpenicillin in *M. sciuri* (*n* = 8), *M. lentus* (*n* = 1), *S. cohnii*, *S. nepalensis,* and *S. saprophyticus* in the absence of β-lactamase encoding genes. Nitrocefin tests confirmed the presence of functional beta-lactamases in all strains encoding a *blaZ* gene while all the *blaZ*-negative strains tested negative with the sole exception of the *S. nepalensis* strain (Fig. 2). In addition, oxacillin resistance was detected in *M. sciuri* (*n* = 15/16, 94%) and *M. lentus* (*n* = 1/2, 50%) and was associated with the presence of chromosomal *mecA1* and *mecA* genes, respectively.

Erythromycin (*n* = 5, 5%) and clindamycin (*n* = 20, 22%) resistances were detected essentially in CoNS. The only exception concerns a multi-drug-resistant *S. pseudintermedius* strain belonging to the ST2363, which presented resistant phenotypes to almost all antibiotics tested including trimethoprim/sulfamethoxazol (TMP/SMX) resistance. For the latter, resistance was linked to the presence of the *dfrG* gene encoding a dihydrofolate reductase. This gene was also found in *S. aureus* (*n* = 7, 15%) and all remaining *S. pseudintermedius*-resistant strains (*n* = 9, 43%). Trimethoprim resistance in the *M. lentus* Dog026 strain was linked to the presence of a *dfrK* gene on a small 6-kbp plasmid, previously described to encode the *tet*(L) gene involved in tetracycline resistance in this strain.

Resistance to aminoglycosides (kanamycin, gentamicin, and streptomycin) was always confirmed by genotypic data (Fig. 2). Streptomycin resistance was associated with the streptomycin nucleotidyltransferase genes *str* (*n* = 2), *ant (6)-Ia,* and *ant (9)-Ia* (*n* = 2) while gentamicin and kanamycin resistances (MICs > 2 and >8, respectively) were linked to the tandem genes *aac(6′)-Ie-aph(2″)-Ia* or *aph(3′)-III* encoding gentamicin and kanamycin acetyl- and phosphotransferases. Several truncated, plasmid-encoded *str* genes were also found in *M. lentus, S. nepalensis,* and *S. saprophyticus* and their presence was associated with susceptible phenotype for streptomycin.

### Virulence-encoding genes in the different *Staphylococcaceae* genomes

**Fig 3:**
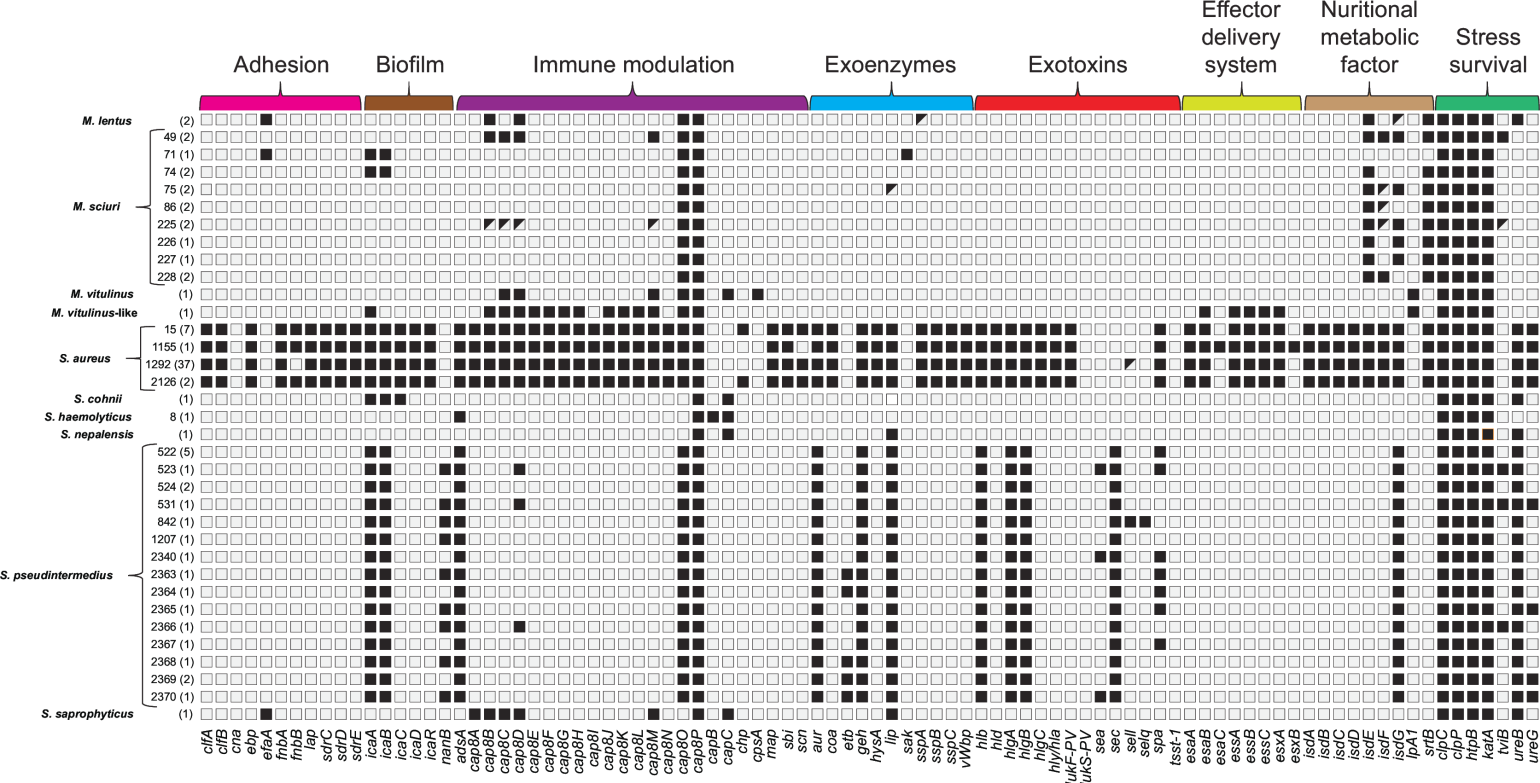
Virulence attributes determined by *in silico* screening of *Staphylococcaceae* strains. The virulence factors (VF) depicted were selected using the VF-database (VFDB) and grouped according to their biological function. Black-filled boxes represent the detection of the corresponding VF in a particular sequence type given the criteria applied. Genes were plotted only if they shared >60% identity over the entire sequence at the nucleotide level with the corresponding VF sequences present in VFDB, which does not exclude the presence of such genes with lower similarity.

We first investigated the overall conservation of virulence factor (VF)-encoding genes present in the VFDB database. As expected, the *S. aureus* strains carried most of the genes investigated including the genes involved in type 8 capsular polysaccharide production (*cap8A-P*), the *ica* (*icaA-D*) and the *isd* (*isdA-G*) operons involved in immune modulation, biofilm formation, and iron uptake, respectively (Fig. 3). From the phenotypic approach, most of these strains (*n* = 46) showed a positive result on the sheep blood agar hemolysis test (only Dog138 was negative). More precisely, 42 *S*. *aureus* strains produced a β-hemolysis while four presented an α-hemolysis (Dog032/ST1292, Dog111/ST1292, Dog119/ST1292, and Dog147/ST15). The *in silico* search for the hemolysis-related genes *hla*, *hlb,* and *hld* gave a uniform and non-ambiguous result. All 47 *S*. *aureus* strains had these three genes in their chromosomes: at >99% identity and 100% coverage for *hla* and *hld*; >99% identity and >81% coverage for *hlb*. As reported previously in the literature, there is not a direct correlation between phenotypic and genotypic hemolytic patterns (24, 25), highlighting the complexity of this multicomponent system. Only minor differences were observed between the different STs present in our data set. For example, some VF-encoding genes were only detected in *S. aureus* belonging to the ST15, ST1155, and ST2126 including a gene coding for the fibronectin-binding protein B (adhesin *fnb*B).

The identification of novel MLST sequence types of *S. pseudintermedius* and *M. sciuri*, as well as the whole-genome sequencing of underrepresented *Staphylococcaceae* species, allowed us to shed light on the presence and conservation of VF-encoding genes in these species. The search for VF-encoding genes in the species *M. lentus*, *M. sciuri*, *M. vitulinus*, *S cohnii*, *S. nepalensis,* and *S. saprophyticus* provided some noteworthy findings (Fig. 3). Interestingly, the *ica* operon consisting of the *icaADBC* genes and involved in biofilm formation presented some genetic variability. The *icaR* gene, a negative regulator of the *ica* operon in *S. aureus*, was absent in all *S. cohnii* and *S. pseudintermedius* strains. On the other hand, upon further investigation of the Prokka and PGAP annotations in this genomic region, an *ica* operon including the *icaR* gene was detected in three *M. sciuri* strains (*n* = 3/16, 19%) belonging to ST71 and ST74. In *S. cohnii*, the four genes *icaABCD* were encoded on a large 48.7 kb plasmid presenting similarities with a plasmid previously reported in *S. cohnii* strain FDAARGOS_744.

The genetic organization of the lipases encoding genes was also different in between species. The gene *lip1*, usually found next to the *ica* operon in *S. aureus*, was found in three copies in all *S. pseudintermedius* strains. Of these three copies, one appeared to be truncated and none was genetically adjacent to the *ica* operon. Such a *lip1* gene was also found in *S. nepalensis* (one copy) *S. saprophyticus* (two copies) and a single *M. sciuri* strain (Dog142). In addition, one copy of the *geh/lip2* gene was also found in all *S. pseudintermedius* strains, as well as in all *S. aureus* strains belonging to the ST15, ST2126, ST1155 and in one *S*. *aureus* strain of ST1292 (Dog112).

The search for exotoxin-encoding genes revealed some peculiarities in our data set. All. *S. pseudintermedius* strains encoded γ-hemolysin genes, namely *hlgA-B*. In addition, the presence of the staphylococcal enterotoxin A precursor (i.e., *sea* gene) was also detected in three *S*. *pseudintermedius* strains (Dog106, 029, 040), whereas the *sec* gene was present in all *S. pseudintermedius* strains. Interestingly, additional copies of both *sec* and *sell* genes were present in one *S*. *pseudintermedius* strain belonging to the ST842 (Dog008). Both genes share high similarity with *S. aureus* orthologous sequences found in VFDB (94.4% and 94.2% of amino acid identity, respectively) and were associated with a complete prophage sequence detected by PHASTER. Finally, at least three hemolysin-encoding genes were detected in the genome of the *S. haemolyticus* strain including two copies of the family protein *hlyC/corC* and one belonging to the hemolysin III family protein.

## DISCUSSION

We investigated the diversity of *Staphylococcaceae* strains colonizing diseased and apparently healthy domestic dogs kept in a shelter in Kenya. The 92 strains isolated and characterized in this study encompass nine validated species and include the two coagulase-positive pathogenic species *S. aureus* and *S. pseudintermedius* besides the coagulase-negative species *M. lentus*, *M. sciuri*, *M. vitulinus*, *S. cohnii*, *S. haemolyticus*, *S. nepalensis,* and *S. saprophyticus*. The latter seven species have been previously reported in dogs from Switzerland (26), the United Kingdom (27), and Germany (28) among others.

Most strains isolated belonged to the species *S. aureus*. Similar results were reported in a Spanish kennel and showed that dogs can be *S. aureus* carriers even if the lineages detected in that study were not necessarily human-specific (29). The *S. aureus* identified in our study belonged to only four STs, ST1292 (CC1; *n* = 37), ST15 (CC15; *n* = 7), ST2126 (CC15; *n* = 2), and ST1155 (*n* = 1), supporting the idea of the spread of specific clones within the shelter. The core genome MLST analysis indicated that 46 out of the 47 *S*. *aureus* strains of this study are likely to originate from only two clones (Fig. S3). This could be explained by an anthropozoonotic event triggered by close contact with staff handling the dogs daily or by a food source. Interestingly, an MLST profiling analysis (Fig. S4) revealed that our ST1292 isolates are closely related to a human *S. aureus* strain isolated in 2015 (ST4707) from a skin wound supporting the idea of a human origin. Novel STs were detected for the species *M. sciuri* (ST225-ST228) and *S. pseudintermedius* (ST522–ST524, ST2363–ST2370). The detection of novel STs was not unexpected, especially considering the huge diversity found in the latter species, and the ecological niche investigated combined with the limited international dog trafficking in sub-Saharan Africa. The presence of *Staphyloccoccaceae* in African dogs was previously investigated, with a focus on clinical samples (21). Overall, two species, namely *S. epidermidis* and *S. pseudintermedius*, were predominantly reported in these studies even if *M. lentus*, *S. haemolyticus,* and *S. cohnii* were also identified on rare occasions. However, strain typing was not performed in any of these studies, which does not allow us to compare the presence of the detected STs in dogs or other hosts in Africa and elsewhere. More recently, we investigated the diversity of *Staphylococcaccae* in dromedary camels in Kenya and Somalia and found the presence of 15 different species including a majority of *S. aureus*, *M. sciuri*, and *S. simulans*, among others (30). However, as expected, none of the STs present in this study were found in our current data set.

Next, we investigated the level of antimicrobial resistance using phenotypic and genotypic data to advise on treatment options for the diseased dogs. Only six out of 92 strains analyzed had a wild-type phenotype to the antimicrobials tested. Most of the resistant phenotypes could be linked to specific resistance genes using a RESFinder-based *in silico* analysis. We did not detect methicillin-resistant strains among the coagulase-positive *S. aureus* and *S. pseudintermedius*, although methicillin-resistant canine coagulase-positive staphylococci were reported from other parts of the continent such as West and South Africa (31, 32). On the other hand, a resistance toward methicillin was observed in our study for the species *M. lentus* and *M. sciuri* conferred by the genes *mecA* and *mecA1*, respectively. These two species were previously reported to accumulate resistance genes in human- and animal-associated strains (33–36). Importantly, our study reports for the first time canine MDR *Staphylococcaceae* in East Africa. MDR coagulase-negative staphylococci, including strains harboring the *mecA* gene, were reported to be present in healthy dogs in other parts of Africa, such as Nigeria (36). Multidrug-resistant strains of our study, resistant to at least three classes of antimicrobials, were among *M. lentus*, *M. sciuri*, *S. aureus*, *S. haemolyticus*, *S. nepalensis,* and *S. pseudintermedius* (Fig. 2). One *S*. *pseudintermedius* strain (ST2363) stood out with resistance against five classes of antimicrobials.

We observed a high manifestation of tetracycline resistance (23%), primarily due to the high prevalence of tetracycline-resistant *S. pseudintermedius* strains (52.3%) compared to *S. aureus* (8.5%) and the CoNS species (25%). As expected, penicillin resistance was very high (Fig. 2). A recent systematic review assessed the overall prevalence of antibiotic-resistant CoPS and CoNS strains isolated from dogs in Africa (37). Our results were generally consistent with the previous reports even if we observed higher penicillin and tetracycline resistance rates in CoPS. Resistance to the other antimicrobials tested (streptomycin, methicillin, kanamycin, gentamicin, erythromycin, and clindamycin) was generally lower in the CoPS species reported in our study. By contrast, a higher prevalence of methicillin-, erythromycin-, and clindamycin-resistant CoNS strains were observed in our study but primarily limited to *M. lentus* (100%, *n* = 2) and *M. sciuri* (100%, *n* = 16). Overall, we report a higher prevalence of MDR *S. pseudintermedius* (10/21, 47%) than previously reported for Africa (37) but a slightly lower prevalence of MDR *S. aureus* strains (8.5% versus 18%).

In addition, low resistance rates to a number of antibiotics tested can be explained by the fact that many of these antimicrobials are not available in Africa due to high costs and disturbed supply chains. The emergence of methicillin-resistant and MDR strains in companion animals in sub-Saharan Africa should be monitored closely because of the potential for zoonotic transmission and the transfer of resistance genes. In particular, the transfer of resistance genes from apathogenic species to medically relevant *S. aureus* and *S. pseudintermedius* strains, likely to be promoted by the inappropriate use of counterfeit or inappropriately stored drugs, requires immediate attention. A recent study showed that wildlife in the urban area of Nairobi carry antimicrobial resistance genes. More specifically, a high prevalence (52%) of *E. coli* strains resistant to many clinically relevant antimicrobials, including nalidixic acid, streptomycin, sulfonamide, tetracycline, and trimethoprim, were found in urban wildlife. The main routes of dissemination of these strains were found to be associated with rodents and seed-eating birds that get in contact with human waste and livestock kept in the close perimeter of habitations (38). Dogs kept in the shelter are likely to have had interactions with human waste and livestock and might have acquired resistant strains that way. A fraction of dogs received antimicrobial treatment at the shelter, which additionally fostered the selection of resistant strains detected in this study. More importantly, this study revealed high resistance prevalences of different *Staphylococcaceae* to tetracycline, penicillin, trimethoprim, and oxacillin. This information will assist in the selection of antimicrobials for the treatment of dogs in the region. This is important since veterinary diagnostic services are absent in many regions of sub-Saharan Africa or are relatively expensive when available compared to industrialized countries.

Virulence-encoding genes were preferentially detected in *S. aureus* strains, which was expected as the VFDB database mainly relies on VF sequences originating from this species. The detailed analysis of VF-encoding genes present in non-*S*. *aureus Staphyloccocaceae* highlighted some particularities and the possibility of horizontal gene transfer (HGT) events in between species. Of note, we detected a *S. pseudintermedius* ST842 strain carrying an extra, phage-associated copy of the *sec* gene encoding the enterotoxin C. This gene shared >95% identity with the *S. aureus sec* gene present in the VFDB database and has been reported to be mainly phage associated with SaPIs (39). A *sell* gene encoding the enterotoxin precursor L, presenting similar high homology with its corresponding *S. aureus* ortholog, was also detected in close vicinity of the *sec* gene. It is very likely that these two genes were acquired together from a single HGT event even if no orthologs on these two genes were found in our data set. We also found that the *ica* operon, responsible for biofilm formation through the production of polysaccharide intercellular adhesin (PIA) (40), was prone to genetic exchange as we detected its presence of the *icaADBC* operon on a large ~48 kb plasmid in *S. cohnii*. A complete *ica* operon has been previously detected on a ~49 kbp plasmid isolated from a bovine methicillin-resistant *S. aureus* strain (41). The reported plasmid encoded several antimicrobial resistance (AMR) and heavy metal resistance genes but did not show compelling homology to the plasmids described in this work. Expression of the *ica* locus was previously shown to be regulated by a variety of environmental factors and the production of PIA is recognized as a key virulence factor in several *Staphyloccoaceae* species including *S. epidermidis* (42). It has been reported that the introduction of the *ica* genes in commensal *S. epidermidis* strains can lead to an invasive phenotype (43). Further investigation would be needed to study the correlation between the presence of the *ica* operon and the invasive capacity of some of the strains present in our data set.

Altogether our study addressed the diversity of canine *Staphylococcaceae* strains found in an animal shelter in Kenya and provide baseline data on their genome content including virulence and antibiotic resistance genes as well as general genome content. We detected nine validated *Staphylococcaceae* species including the pathogenic coagulase-positive species *S. aureus* and *S. pseudintermedius*. While *S. aureus* is likely to be acquired from humans or a food source, the *S. pseudintermedius* strains represent 11 novel STs, highlighting the diversity of such a geographical niche for a species associated with the microbial flora of the tested dogs. Both coagulase-negative and coagulase-positive *Staphylococcaceae* investigated contained subsets of resistance genes and VF-encoding genes, which might constitute a reservoir for other bacteria and pose a threat of human health in case of zoonotic transmission.

## MATERIALS AND METHODS

### Collection of specimens and isolation of *Staphylococcaceae*

Diagnostic specimens from all dogs (*N* = 167) housed at the animal shelter were collected on the 27th June 2015 in Nairobi, Kenya by veterinarians using the TRANSWAB® Amies swabs. Nasal specimens were collected by inserting the swabs up to 10 cm into the nasal cavity of the animals, which were restrained without sedation for the procedure. Wound infections were also swabbed when present. The *Staphylococcaceae* strains were isolated at the laboratories of the International Livestock Research Institute (ILRI) using standard methods without selective enrichment (44). Briefly, each swab was streaked on Baird Parker Agar (Carl Roth) plates and incubated at 37°C overnight. One to two suspicious colonies per plate were picked, expanded in LB media (Carl Roth), and stored as glycerol stocks at −80°C before being shipped for further characterization to the Institute of Veterinary Bacteriology (IVB) in Switzerland. Strains were streaked onto Trypticase Soy Agar (TSA-B; Becton Dickinson) with 5% sheep blood (TSA-B; Becton Dickinson) and incubated at 37°C overnight. Species designation was assigned using MALDI-TOF MS (Bruker) as previously reported (30) followed by metabolic phenotyping (see below). Data on strains investigated in this study are provided in Dataset S1.

### Phenotypic characterization and antimicrobial susceptibility testing

Phenotypic metabolic testing was done using the VITEK 2 GP identification card *via* the automated VITEK 2 COMPACT system (bioMérieux). Samples were prepared according to the manufacturer’s recommendations using overnight cultures grown on TSA-B at 37°C. The minimal inhibitory concentration (MIC) of 12 antibiotics, that is, benzylpenicillin (MIC >0.125 µg/mL), oxacillin (MIC ≥4 µg/mL for *S. aureus* and MIC ≥1 µg/mL for non*-S. aureus* strains), cefoxitin (MIC >4 µg/mL), kanamycin (MIC >8 µg/mL), gentamicin (MIC >2 µg/mL), streptomycin (MIC >32 µg/mL), trimethoprim (MIC >4 µg/mL), trimethoprim/sulfamethoxazole (ratio 1:20) (MIC ≥2/38), doxycycline (MIC >0.5 µg/mL), tetracycline (MIC >2 µg/mL), erythromycin (MIC >2 µg/mL), and clindamycin (MIC >0.25 µg/mL) was determined by broth microdilution in cation-adjusted Mueller-Hinton Broth (CAMHB) using SENSITITRE EUST2 and COMPAN1 plates (ThermoScientific). The MIC was interpreted using the European Committee on Antimicrobial Susceptibility Testing (EUCAST) resistance breakpoint except for oxacillin and trimethoprim/sulfamethoxazole for which the CLSI recommendations for coagulase-negative species were used (CLSI Supplement M100. Clinical and Laboratory Standards Institute; 2021).

In addition, the presence of functional β-lactamase activity was assessed on all strains using the BBL Dryslide Nitrocefin kit according to the manufacturer’s recommendations. Briefly, strains were grown overnight on 5% sheep blood agar plates and induced using Oxoid Penicillin G antimicrobial susceptibility discs. Results were recorded after a 30-min incubation at room temperature.

### Next-generation sequencing

Genomic DNA was isolated using a phenol:chloroform:isoamyl alcohol (25:24:1) extraction method as previously described (30). The genomic DNA quality and the quantity was measured by QuDye dsDNA HS Assay Kit (Invitrogen) on a Qubit 3 Fluorometer (Invitrogen). Library preparations and sequencing were done on a PacBio Sequel II instrument (Pacific Biosciences) at the Lausanne Genomic Lausanne Genomic Technologies Facility (GTF) as previously described (45).

### Genome assembly, annotation, and *in silico* characterization of extrachromosomal DNA

All bioinformatics analysis was performed on local Ubuntu machines (18.04LTS and 20.04LTS) and servers of the IBU Linux Cluster from Bern, using custom bash, R, and Perl scripts. Default parameters were used for all software unless stated otherwise. Genome assemblies and circularization were done from the PacBio reads using Flye v2.8 (46) and Circulator (47), respectively. Circularized genomes were polished with three rounds of Arrow (SMRTLink8 package) and rotated with a custom script to the first nucleotide of the *dnaA* gene. Sequences were annotated using Prokka 1.13 (48) and Prodigal (49), while tRNA prediction was performed using ARAGORN (50) and RNAmmer software (51). PHASTER (52) was used to scan chromosomes to locate prophage sequences. The non-chromosomal circularized contigs were screened for known plasmid and phage sequences using PlasmidFinder (v2.1.1) (53) and PHASTER, respectively. BlastN searches (https://blast.ncbi.nlm.nih.gov/) were also performed when no hits were detected using the two previous methods. Prokka, eggNOG-mapper (2.1.6) (54), and RAST (55) annotation tools were used to investigate further functional signatures of these putative plasmid and phage molecules. The canine *S. aureus* core genome and its alignment were built with Roary (56, 57) pipeline, the phylogenetic tree was inferred with IQ-TREE2 (v2.2) (58–60) and plotted with FigTree (v1.4.4) (http://tree.bio.ed.ac.uk/software/figtree/). Additional *in silico* investigations were performed using the OpenGenomeBrowser (OGB) platform (61) and a local database harboring all *Staphyloccocaceae* genomes sequenced in this study as well as high-quality genomes retrieved from GenBank.

### Generation of minimum spanning (MS) trees using MLST) data

MLST was performed for the *Staphylococcaceae* species from our data set that has schemes available on the PubMLST database (62), namely *M. sciuri*, *S. aureus*, *S. haemolyticus,* and *S. pseudintermedius*. Allele sequences were retrieved from the genome sequences and Multilocus Sequence Typing (MLST) profiles of strains were uploaded to the respective databases. The minimum spanning trees (MST) were inferred using Bionumerics v8.1.1 (Biomérieux) as previously described (63).

### Detection of genes encoding antimicrobial resistance and virulence factors

The *in silico* search for genes conferring antimicrobial resistance was performed using ResFinder 4.0 (64). Virulence factors-encoding genes were identified with ABRicate (https://github.com/tseemann/abricate) using the virulence factor database (VFDB, http://www.mgc.ac.cn/VFs/main.htm) (65). Genes plotted had identity scores above 60% at the nucleotide level. SCC*mec*Finder (https://cge.food.dtu.dk/services/SCCmecFinder/) was used to screen for *mecA*-associated SCC*mec* cassettes (66). The *in silico* characterization of the replicative proteins found on plasmids was done using PlasmidFinder (67). The presence of insertion sequences (IS) and mobilization sequences (i.e., relaxases and origin of transfer) were done using ISfinder (68) (https://isfinder.biotoul.fr/) and *ori*Tfinder (69) (https://tool-mml.sjtu.edu.cn/oriTfinder/contact.html), respectively.

## Data Availability

Genomic data have been deposited under the NCBI project number PRJNA942599.

## SUPPLEMENTAL MATERIAL

Dataset S1 spectrum.02924-23-s0001.xlsx Metadata, phenotypic and resistance data as well as genomic data of the canine *Staphylococcaceae*.

Figure S1 spectrum.02924-23-s0002.pdf Minimum spanning trees of *Staphylococcaceae* with respect to the geographic origin of the isolated strains. Minimum Spanning Tree (MST) based on multilocus sequence typing (MLST) data were built based on (A) 23 *Mammaliicoccus sciuri* strains, (B) 611 *Staphylococcus aureus* strains, and (C) 25 *Staphylococcus pseudintermedius* strains. The MLST data were downloaded from PubMLST and the trees were built with Bionumerics v8.1.1. The sequence type (ST) numbers are displayed in black, while gray numbers indicate allele differences between the STs. The geographical origin of the samples is displayed using the color code depicted in the legend.

Figure S2 spectrum.02924-23-s0003.pdf Mobilome of the canine *Staphylococcaceae* strains. (A) Plasmids found and their relative GC content. Dots represent circularized extrachromosomal DNA sequences identified as plasmids by PlasmidFinder. A continuous gray line shows the mean relative deltaGC value and the dashed gray lines indicate two standard deviations (2*SD) from the relative deltaGC mean value. (B) Prophage sequences identified by PHASTER. Intact (score >90), questionable (score 70-90) and incomplete (score <70) putative prophages are displayed.

Figure S3 spectrum.02924-23-s0004.pdf Core genome phylogenetic tree of the canine *S. aureus* of this study. The core genome and its alignment were built with Roary pipeline. The phylogenetic tree was inferred with IQ-TREE2 software and plotted with FigTree. The *S. aureus* type strain DSM20231T was included as outgroup.

Figure S4 spectrum.02924-23-s0005.pdf Minimum spanning tree of African *S. aureus* strains isolated between 2013 and 2017 and deposited in the PubMLST database. The tree was built with Bionumerics v8.1.1. The origin of the samples is displayed using the color code depicted in the legend. A, B and C are zoom-ins of regions harboring canine STs described in this study. The sequence type (ST) numbers are indicated in black, while gray numbers indicate allele differences between the STs.

ASM does not own the copyrights to Supplemental Material that may be linked to, or accessed through, an article. The authors have granted ASM a non-exclusive, world-wide license to publish the Supplemental Material files. Please contact the corresponding author directly for reuse.
