# Supplementary material for: Canine Staphylococcaceae circulating in a Kenyan animal shelter
Source: Microbiol Spectr. 2024 Jan 11;12(2):e02924-23. doi: 10.1128/spectrum.02924-23 (PMC10846116; doi:10.1128/spectrum.02924-23)
Supplement: Figure S1 — Minimum spanning trees of Staphylococcaceae with respect to the geographic origin of the isolated strains. Minimum Spanning Tree (MST) based on multilocus sequence typing (MLST) data were built based on (A) 23 Mammaliicoccus sciuri strains, (B) 611 Staphylococcus aureus strains, and (C) 25 Staphylococcus pseudintermedius strains. The MLST data were downloaded from PubMLST and the trees were built with Bionumerics v8.1.1. The sequence type (ST) numbers are displayed in black, while gray numbers indicate allele differences between the STs. The geographical origin of the samples is displayed using the color code depicted in the legend. [file spectrum.02924-23-s0002.pdf]

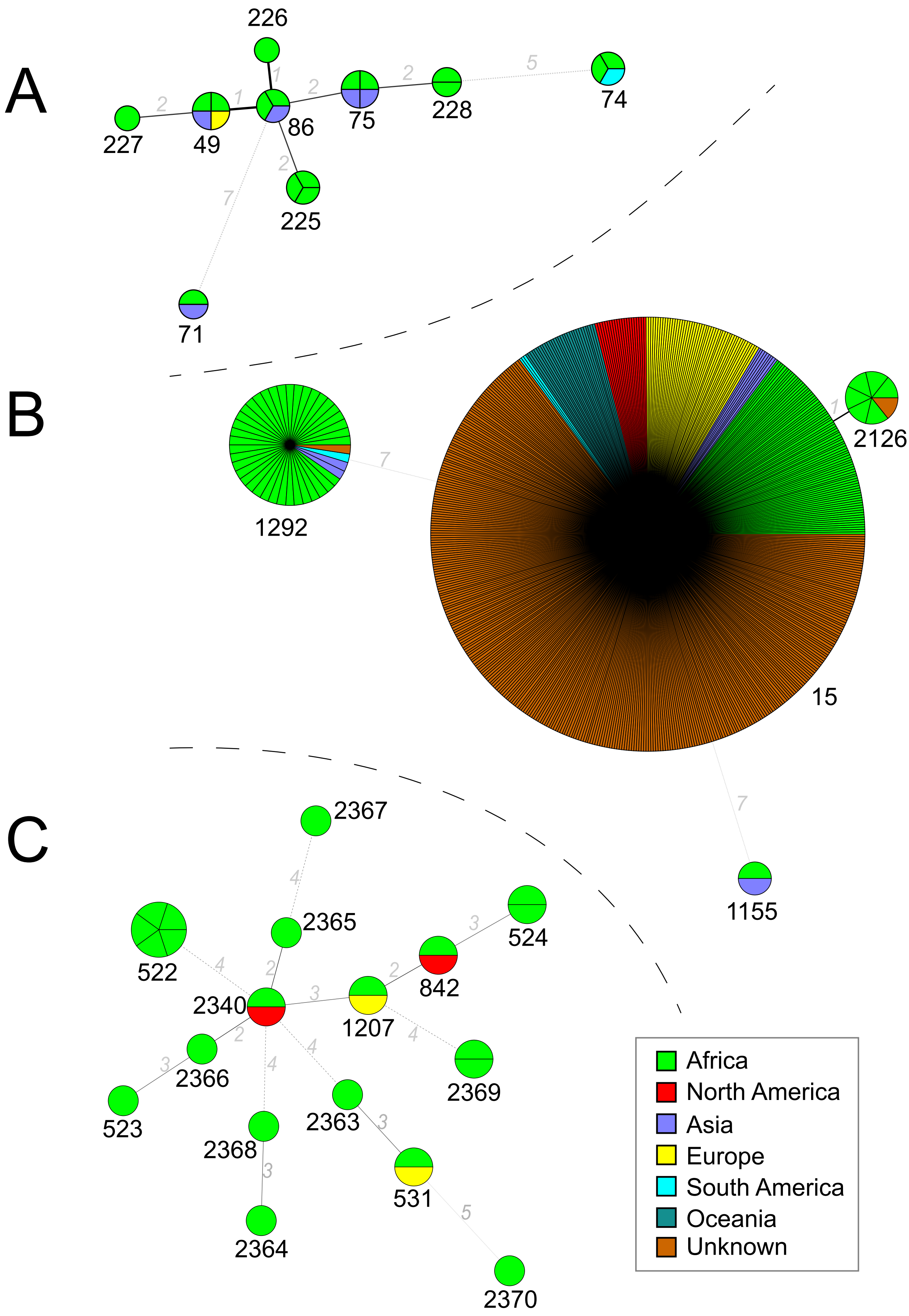

Figure S1. Minimum spanning trees of *Staphylococcaceae* with respect to the geographic origin of the isolated strains. Minimum Spanning Tree (MST) based on multilocus sequence typing (MLST) data were built based on **(A)** 23 *Mammaliicoccus sciuri* strains, **(B)** 611 *Staphylococcus aureus* strains and **(C)** 25 *Staphylococcus pseudintermedius* strains. The MLST data were downloaded from PubMLST and the trees were built with Bionumerics v8.1.1. The sequence type (ST) numbers are displayed in black, while grey numbers indicate allele differences between the STs. The geographical origin of the samples is displayed using the colour code depicted in the legend.
