# Supplementary material for: Canine Staphylococcaceae circulating in a Kenyan animal shelter
Source: Microbiol Spectr. 2024 Jan 11;12(2):e02924-23. doi: 10.1128/spectrum.02924-23 (PMC10846116; doi:10.1128/spectrum.02924-23)
Supplement: Figure S2 — Mobilome of the canine Staphylococcaceae strains. (A) Plasmids found and their relative GC content. Dots represent circularized extrachromosomal DNA sequences identified as plasmids by PlasmidFinder. A continuous gray line shows the mean relative deltaGC value and the dashed gray lines indicate two standard deviations (2*SD) from the relative deltaGC mean value. (B) Prophage sequences identified by PHASTER. Intact (score >90), questionable (score 70-90) and incomplete (score <70) putative prophages are displayed. [file spectrum.02924-23-s0003.pdf]

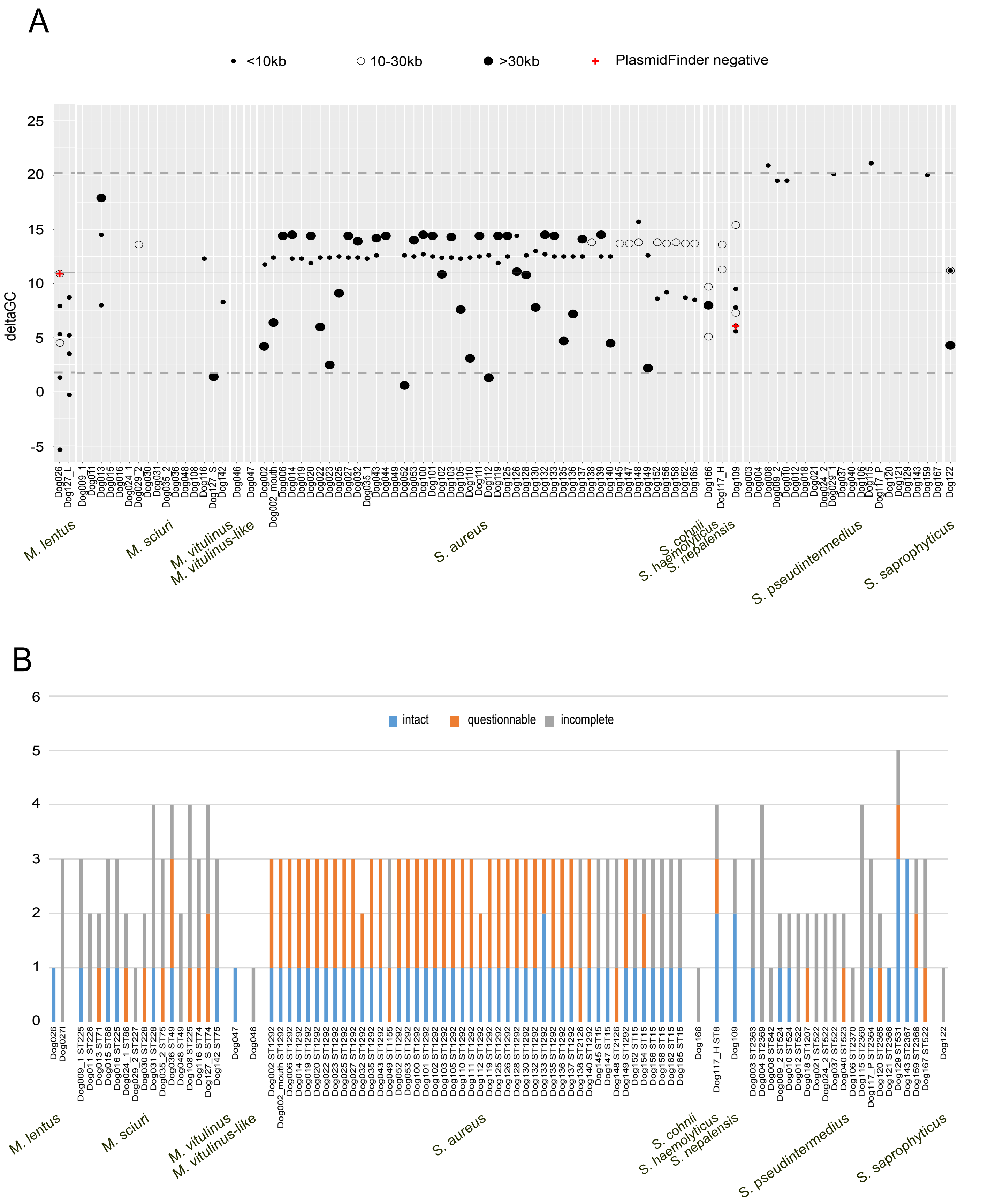

Figure S2. Mobilome of the canine *Staphylococcaceae* strains. (A) Plasmids found and their relative GC content. Dots represents circularized extrachromosomal DNA sequences identified as plasmids by PlasmidFinder.
A continuous grey line shows the mean relative deltaGC value and the dashed grey lines indicate two standard deviation (2*SD) from the relative deltaGC mean value. (B) Prophage sequences identified by PHASTER. Intact (score >90), questionable (score 70-90) and incomplete (score <70) putative prophages are displayed.
