# Supplementary material for: Canine Staphylococcaceae circulating in a Kenyan animal shelter
Source: Microbiol Spectr. 2024 Jan 11;12(2):e02924-23. doi: 10.1128/spectrum.02924-23 (PMC10846116; doi:10.1128/spectrum.02924-23)
Supplement: Figure S3 — Core genome phylogenetic tree of the canine S. aureus of this study. The core genome and its alignment were built with Roary pipeline. The phylogenetic tree was inferred with IQ-TREE2 software and plotted with FigTree. The S. aureus type strain DSM20231T was included as outgroup. [file spectrum.02924-23-s0004.pdf]

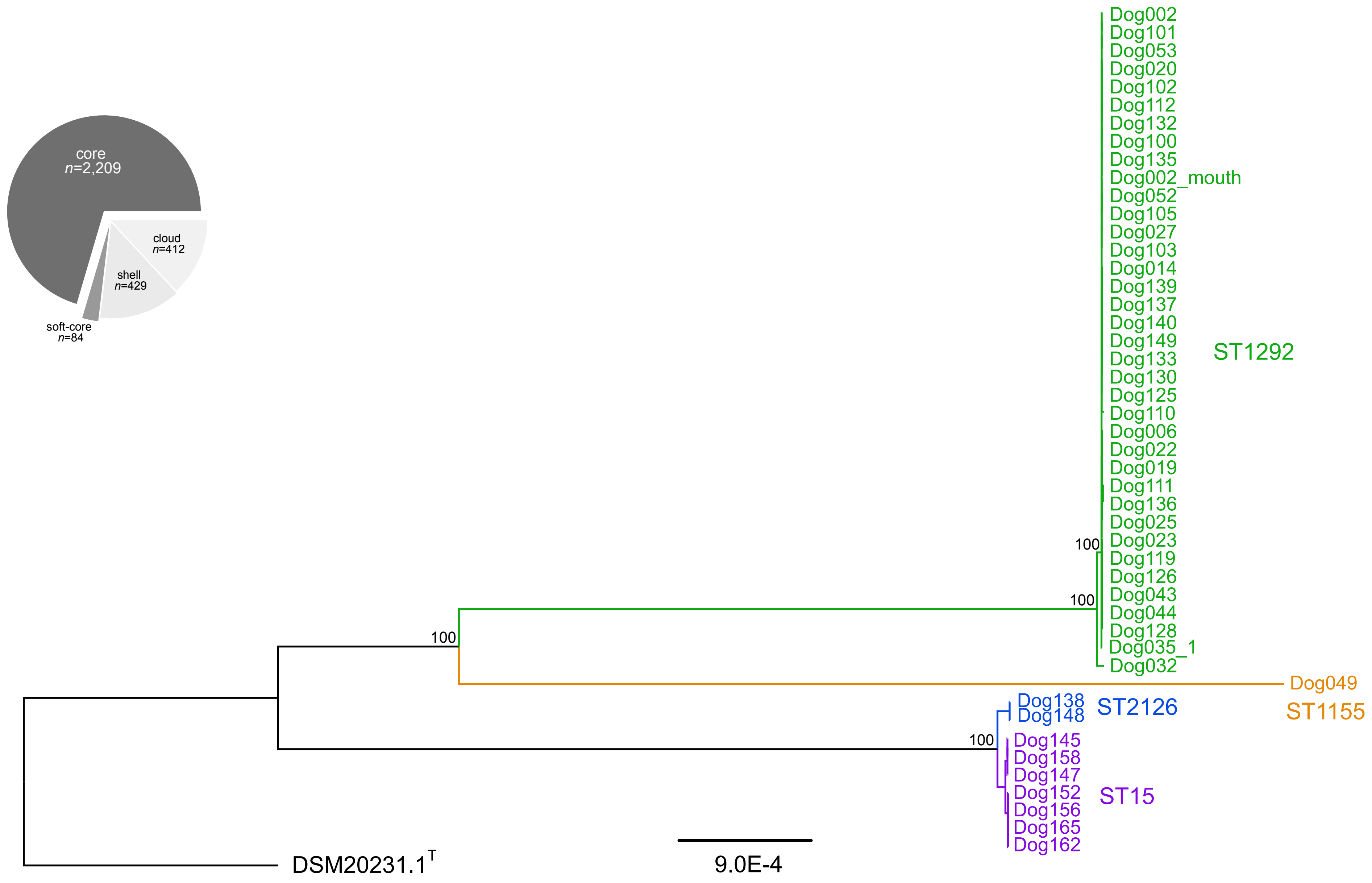

Figure S3. Core genome phylogenetic tree of the canine *S. aureus* of this study. The core genome and its alignment were built with Roary pipeline. The phylogenetic tree was inferred with IQ-TREE2 software and plotted with FigTree. The *S. aureus* type strain DSM20231^{T} was included as outgroup.
