# Supplementary material for: Canine Staphylococcaceae circulating in a Kenyan animal shelter
Source: Microbiol Spectr. 2024 Jan 11;12(2):e02924-23. doi: 10.1128/spectrum.02924-23 (PMC10846116; doi:10.1128/spectrum.02924-23)
Supplement: Figure S4 — Minimum spanning tree of African S. aureus strains isolated between 2013 and 2017 and deposited in the PubMLST database. The tree was built with Bionumerics v8.1.1. The origin of the samples is displayed using the color code depicted in the legend. A, B and C are zoom-ins of regions harboring canine STs described in this study. The sequence type (ST) numbers are indicated in black, while gray numbers indicate allele differences between the STs. [file spectrum.02924-23-s0005.pdf]

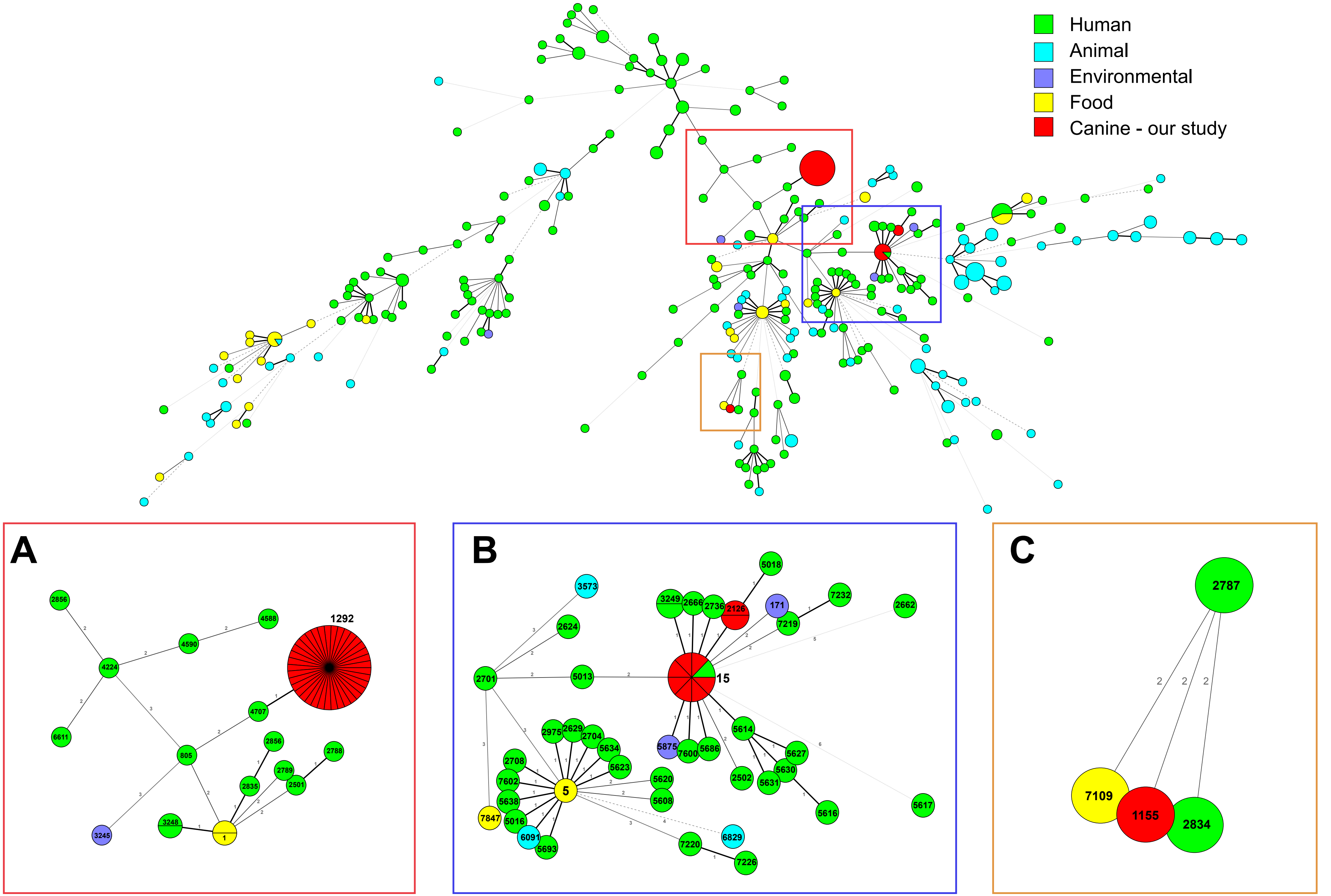

Figure S4. Minimum spanning tree of African *S. aureus* strains isolated between 2013-2017 and deposited in the PubMLST database. The tree was built with Bionumerics v8.1.1. The origin of the samples is displayed using the color code depicted in the legend. A, B and C are zoom-ins of regions harboring canine STs described in this study. The sequence type (ST) numbers are indicated in black, while grey numbers indicate allele differences between the STs.
